# An initial evaluation of a family-based approach to weight management in adolescents attending a community weight management group

**DOI:** 10.1111/j.1365-277X.2012.01277.x

**Published:** 2012-07-27

**Authors:** A Avery, C Pallister, J Allan, J Stubbs, J Lavin

**Affiliations:** Nutrition and Research Team at Slimming WorldAlfreton, Derbyshire, UK

**Keywords:** behaviour change, commercial slimming organisation, family, obesity, weight management in adolescents

## Abstract

**Background:**

Family-based approaches are recommended for the prevention and management of childhood obesity. Given the large numbers of obese children, scalable practical solutions are required. The present study evaluated a family-based national programme that aimed to empower adolescents to adopt healthier lifestyles.

**Methods:**

Group facilitators supporting more than six young members (11–15 years) participated in the study. A questionnaire was designed to determine the characteristics of the adult attending with the adolescent, any health professional recommendations given and the young member’s integration within traditional adult weight management groups. Data on measured height and weight [and calculated body mass index (BMI)], sex and attendance were collated from member’s records.

**Results:**

Questionnaires were completed by 22 facilitators (15% response rate), representing data for 128 young members with complete weight data available for 106. All members had a joining BMI > 91st centile, with 68% >98th centile. The mean (SD) number of weeks attended was 12.5 (8.1), with 19% (20) having attended for more than 20 weeks with 62% still attending. A mean (SD) BMI *Z*-score change of 2.49 (0.72) to 2.27 (0.74) was achieved (*P* < 0.001). The relationship of the adult supporter to the young member was varied, with 62% either already members or joining alongside their daughter/son. Limited guidance was provided by health professionals before or during attendance. Facilitators were comfortable about the age mix within groups.

**Conclusions:**

The community weight management organisation studied takes a family-based approach and successfully supports young members to manage their weight.

## Introduction

Childhood obesity is one of the most significant health challenges currently facing the UK because it is difficult to treat and is increasing in prevalence (Department of Health, 2008). Presently, 36% of boys and 34% of girls, aged 11–15 years, are overweight or obese (NHS Information Centre, 2010). It is predicted that, as a result of our increasingly obesogenic environment, the prevalence will rise to almost 70% of girls and 55% of boys by the year 2050 (Foresight, 2007).

Obesity affects not only the current physical and psychological health of the child, but also future health because unaddressed obesity tends to persist into adulthood (Singh *et al.*, 2008). The physical consequences of childhood obesity include an increased risk of type 2 diabetes diagnosed in childhood, and a predisposition to cardiovascular risk factors, such as hypertension, dislipidaemia and hyperinsulinaemia (Srinivasan *et al.*, 1996; Figueroa-Colon *et al.*, 1997; Malecka-Tendera & Molnar, 2002).

Overweight and obese children show greater signs of psychological distress than their slim counterparts and are more likely to have poor self-esteem, be depressed, have body image dissatisfaction, be perceived as unattractive and present with disordered eating or bulimia, all of which may have long-term effects on the child, continuing into adulthood [NICE, Department of Health, 2006; Department of Health, 2008; Scottish Intercollegiate Guidelines Network (SIGN), 2010].

Current UK strategies to tackle the rising prevalence of obesity focus on prevention and management in children; for example, Healthy Weight, Healthy Lives (Department of Health, 2008). As such, the Government has set a target to stem the rise in prevalence of obesity in children by 2020 (Public Service Agreement 12) and launched family-based initiatives (Department of Health, 2009). Current guidelines focus on lifestyle, physical activity and behavioural change strategies that account for the child’s preferences, risk factors/co-morbidities, social circumstances and previous treatment results. These include the promotion of habitual physical activity, the reduction of sedentary behaviour (including time spent in front of a screen) and healthier eating practices (NICE, Department of Health, 2006; SIGN, 2010). Family-based approaches are recommended because there is continuing evidence of an association between overweight and obesity in children and parental overweight and obesity (Davison & Birch, 2001). Also, many young people are unable to make changes without the support of an adult family member (SIGN, 2010)

There are a small number of evaluated programmes available to help obese children to lose weight. The MEND (Mind, Exercise, Nutrition and Do it) programme is designed to support 7–13 year olds with managing their weight. In a randomised controlled study, Sacher *et al.* (2010) reported a mean body mass index (BMI) *Z*-score change of −0.24 after 6 months in children attending the educational and activity-based programme.

The WATCH IT programme was developed to address the needs of obese children from disadvantaged communities in Leeds. The results of the pilot phase showed significant weight change in the 94 children, mean age 12.2 years, attending the community service, with a signifiant BMI *Z*-score change being achieved by the teenage subpopulation (Rudolf *et al.*, 2006). The Scottish Childhood Overweight Treatment Trial (SCOTT) involved randomising overweight children (aged 5–11 years) to either normal dietetic support or to the intervention, which was delivered by experienced paediatric dietitians offering a family-centred behavioural support programme. A significant change in BMI *Z*-score was observed for both the control and the intervention groups (Hughes *et al.*, 2008). In addition, residential weight management camps have been well evaluated, with a BMI *Z*-score change of −0.25 in 61 teenagers with a mean age of 14.1 years (Barton *et al.*, 2004), although there are no published data available for the weight management groups that Carnegie have introduced for obese children.

The present study aimed to evaluate a national family-centred weight control programme run within a commercial weight management group setting where young people aged 11–15 years were able to attend the weekly group sessions at no charge, although with the proviso that they attend with a supporting adult. The programme focused on facilitation of behaviour change through the consumption of lower energy dense foods, healthier snacks and increases in physical activity. To date, there has been no evaluation of a mixed age weight management programme where young people attend with a supporting adult.

## Materials and methods

### Weight management programme

A family-based group programme, Family Affair, was launched in January 2006. This aimed to support adolescents (aged 11–15 years) with their weight management via engagement of the whole family in adopting healthier lifestyle habits. All group facilitators were provided with additional training emphasising key nutritional points pertinent to adolescents. In an 8-month period, 4704 young people aged 11–15 years had or were currently accessing group support. Facilitators of groups with 6–18 young members were identified and asked to take part in the survey.

### Questionnaire design and distribution

A questionnaire was developed for completion by group facilitators and was piloted with a small number. The questionnaire aimed to identify which adults attended with the young members, whether this adult was already a member of the group or if they joined as a member at the same time as the young person and, if so, whether they joined to also receive weight management support themselves or just to support their child. Information was also requested on whether other immediate family members attended the group and what recommendation was provided to the facilitator by the supporting health professional (e.g. the level of support required in terms of whether the young person should be weighed or not, whether they would benefit just from lifestyle advice, or direction as to whether the young person should be losing weight or just halting weight gain).

Also, the feelings of the group facilitator about having young people as part of their group were assessed via two questions included in the questionnaire and an open question inviting qualitative comments.

Questionnaires were distributed via the group facilitators and, once completed, returned to the principal investigator for analysis by post. Where returned data were incomplete, every effort was made to retrieve missing data.

### Demographics and anthropometrics

Age, sex, weight and height measures were routinely collected in the groups and recorded. Young members were weighed using bespoke Seca scales (Seca GmbH & Co. KG., Hamburg, Germany) accurate to 0.23 kg and height was measured using a stadiometer by their healthcare professional. From the raw data collected, age-appropriate individual start and end BMI were then calculated by the principal investigator.

### Statistical analysis

The results are presented using simple descriptive statistics [mean (SD)], calculated using microsoft excel 2003 (Microsoft Corp., Redmond, WA, USA). Where appropriate, *Z*-scores were used to describe the distribution of BMI relative to reference values (Cole & Green, 1992). The lead researcher analysed the qualitative data by thematic content analysis. A rigorous process was employed that included careful transcription of the reported information. This process resulted in recurring themes that were not predefined, but which best reflected participants’ perceptions. The data were analysed using a cyclical, reflective process (Carter, 2004). Discussion of the themes and sub themes, including a second researcher, was undertaken and agreed upon. Analysis of the data involved the processes of data reduction, data display and data complication. These three processes involved selecting and focusing the data, and data organisation followed by data construction to draw conclusions (Miles & Huberman, 1994). Identified themes that best support the quantitative data are presented.

Ethical approval was not sought because the focus is on service evaluation, in accordance with the NRES (2009) guidance differentiating between audit, service evaluation and research.

## Results

**Figure 1:**
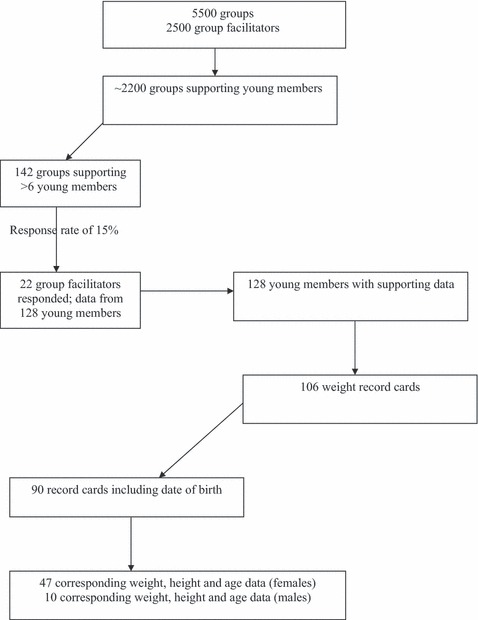
Flowchart summarising recruitment of the study population.

The response rate was 15%, with 128 (of 852) completed questionnaires being returned by 22 group facilitators. Complete data on weight, height and age were available on 47 females and 10 males (Fig. 1). All members had a joining BMI >91st centile, with 68% >98th centile. Starting weights are described in Table 1.

**Table 1:** Body mass index (BMI) centile range of participants on joining the group

	BMI centile range of participants on joining		
	
	91st–98th	98–99.6th	>99.6th
Number of girls	16	18	13
Number of boys	2	3	5

### Attendance

The mean (SD) number of weeks attended was 12.5 weeks (8.11), with 20 out of the 106 having attended for more than 20 weeks, 37 having attended for more than 10 weeks, and 66 (62%) still attending the weekly group.

### Weight changes

**Figure 2:**
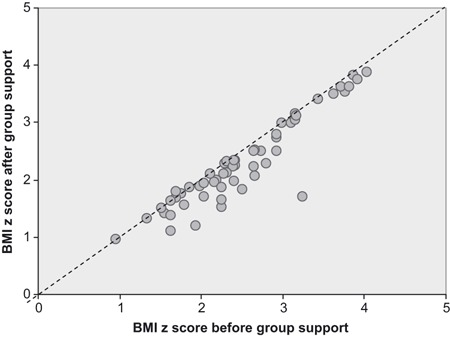
Changes in body mass index (BMI) *Z*-score achieved through the attendance at a community weight management group (*n* = 57).

There was a wide range of weight changes (a gain of 2.0 kg to a weight loss of 10.5 kg). The mean (SD) weight change was −3.0 kg (9.5). The mean BMI change was −1 (29.3 to 28.3 kg m^−2^) This represented a mean (SD) BMI *Z*-score change of 2.49 (0.72) to 2.27 (0.74) at the end of the study period, with this change being statistically significant (*P* < 0.001). BMI *Z*-score changes are shown in Fig. 2.

### Supporting family members

The majority of young members attended group with their mother (84.4%), with 1.5% attending with their father and 3.9% with both parents. The remaining 10.2% attended with a grandparent or other supporting adult.

Sixty-two percent of supporting family members were already members themselves, accessing group support to lose weight. Of the 48 adults who were not already a member, 38 (79.2%) joined with the young person to obtain weight management support for themselves too.

**Figure 3:**
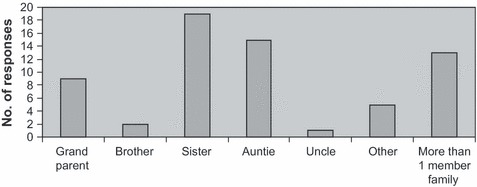
Immediate family members also attending the community weight management support group.

Sixty-four (60%) of the responses highlighted that other immediate family members, in addition to the young member’s supporting parent, also attended the weekly weight management group (Fig. 3).

### Guidance by a health professional

Only 76 of the 128 (59%) young members were given guidance by a health professional, usually their general practitioner, with respect to the appropriate level of support required in terms of whether the young person should be weighed or not or whether they would benefit just from lifestyle advice, or direction as to whether the young person should be losing weight or just halting weight gain. Where health professional guidance was given, 27.6% of the health professional recommendations were to aim to slow current weight gain, 10.5% to maintain current weight, 40.8% to lose weight, and 21.1% for healthy eating and activity advice only with no need to weigh the young person.

### Feedback from group facilitators

Analysis of how the group facilitators felt about having young members in their weekly weight management groups suggests that 77.7% felt either ‘very comfortable’ or ‘comfortable’ about welcoming and supporting them. Nineteen percent said that they were ‘okay’ with the introduction of the family-based group programme and only 3.4% felt ‘uneasy’. No-one stated that they felt ‘very anxious’ about having young people in the group environment. With respect to how well the 11–15 year olds had fitted into the group, 64.9% of the group facilitators felt ‘very well’, 16.7% felt ‘quite well’, 16.7% felt ‘okay’ and only 1.8% felt ‘not at all well’. There were a number of qualitative comments to this last question, which alluded, for example, to how well the young person participated in the group environment:

‘female M joins in with the group really well, comes up with different recipes and ideas’, ‘female R was delighted with her weight loss and it totally changed her, giving her so much confidence, and now she no longer attends hopefully she will have a better understanding of healthier eating’

However, there were also general comments regarding the importance of the family support and concern that lack of support from some parents could sabotage the young person’s weight management journey, either through their inability to commit to supporting their teenager or because of their own personal issues with weight management and inability to lose weight. Examples of this type of comment included: ‘I felt mum lost interest’, ‘mum never had time to cook or to be much help’, ‘mum herself put on weight and then stopped coming’, ‘her mum won’t make time to stay to each group – always too busy’, and:

‘female K is a very sensible girl and knows about healthy eating and she loves cooking and experimenting with foods; it’s K’s mum who is up and down with her weight loss and sometimes causes the problem with female K’ (quote from a group facilitator)

An additional observation made by the principal investigator, from both the qualitative data and the individual weight record cards, was that many of the young people found weight management more difficult over a long school holiday period. Approximately 65% of those young people who were members over the summer vacation gained a small amount of weight, whereas other young members (8%) did not return to the group following a holiday and/or during the holiday period.

## Discussion

The family-based group programme for young members was introduced following consultation with adult members, particularly parents, a review of current literature on the effectiveness of weight management in this age group and consultation with an advisory panel, including nutritionists, dietitians, medical experts and psychologists. Given the well documented increase in prevalence of excess weight in the adolescent population (National Health Service Information Centre, 2010), it was considered appropriate to investigate the possibility of providing support for 11 and 15 year olds within existing groups free of charge. The proviso was that young people should attend with the parent or guardian who was responsible for providing the majority of their food, and have the support of a health professional who would be involved in monitoring and weight management goal setting.

The mean weight loss of 3.0 kg represents a range of weight changes, as one would expect from an age group that represents young people at differing pubertal stages. Only nine of the 106 young people who participated (and for whom weight records were available) actually gained any weight during the study period, with the greatest weight gain being 2.0 kg. The BMI *Z*-score change of −0.22 is very similar to the change of −0.24 reported by Sacher *et al.* (2010) in the randomised control trial using the MEND programme as the intervention. This was also similar to the BMI *Z*-score change of −0.13 achieved by the teenagers attending the health trainer-led Watch IT groups in Leeds (Rudolf *et al.*, 2006) and to the change of −0.25 achieved by teenagers attending a residential weight management programme (Barton *et al.*, 2004).

Where complete data are available, the findings suggest that all young people accessing the group support were initially above the 91st and many above the 98th centile, and were therefore classified as clinically overweight or very overweight (Child Growth Foundation, 2009). Thus, an interesting discussion point is whether, without some weight loss, these young people would be able to achieve a healthy body weight during the transition from puberty to adulthood. In the past, health professionals have tended to ‘sit on the fence’ and suggest halting weight gain as being appropriate for this age group and that ‘hopefully’ they will ‘grow out’ of their obesity. Given the increasing rise in obesity and the fact that all of those with a complete data set available would be classified as clinically overweight or obese (NICE, Department of Health, 2006), some weight loss should be considered as appropriate; after puberty, projected linear growth is unlikely to result in a healthy and age-appropriate BMI if weight remains stable.

Community weight management organisations have been criticised as only attracting females and, indeed, for this particular organisation, 95% of the adult group members are female. However, 20% of the young people in this study population were male, with some being very successful in managing their weight. One male had lost 10.5 kg at the end of the study period and continued to attend the weekly groups until he had achieved his own ‘happy’ weight, despite the fact that he had to start paying a weekly fee on reaching the age of 16 years. Nineteen out of the twenty males with complete data showed reductions in BMI at the end of the study period, with the remaining one male showing no change.

The actual mean number of weeks attended by the young people is unknown because 62% were still attending at the end of the evaluation period. However, 20 had attended for more than 20 weeks and 37 had attended for more than 10 weeks. Young people in this age-range do not generally commit to attending a weekly group for this length of time unless they feel they are benefitting in some way and certainly they would need to feel comfortable within the group environment and with other group members (Cohen & Emanuel, 1998). Other weight management programmes available for this age group, such as MEND and Carnegie Weight Management clubs, tend to be for a fixed period of 10–12 weeks with limited follow-up. However, the data presented in this evaluation would suggest that young people may wish for a longer period of group support and there may be benefits from having a flexible approach where they can attend groups for as long as they feel appropriate.

The evidence base for the management of childhood obesity (NICE, Department of Health, 2006) emphasises the need for family support and some child weight management programmes just target the parents. SIGN guidance recommends that treatment programmes for managing childhood obesity should incorporate behaviour change components, be family-based, involving at least one parent/carer, and aim to change the whole family’s lifestyle. Programmes should target decreasing overall dietary energy intake, increasing levels of physical activity and decreasing time spent in sedentary behaviours (screen time).

In this programme, 11–15 year olds are welcomed into groups free of charge, with the proviso being that they attend with a supporting adult family member (ideally the major food provider). Although the majority (84%) of those supporting the young person turned out to be the mother, other family members were important in this role and, given the structure of some families, this is important to respect. Ideally, as the qualitative data suggests, this supporting family member needs to be someone who has time and enthusiasm to commit to further empowering the young person. Perhaps, unsurprisingly, the majority (62%) of those adults supporting the young person were already members of the group themselves and accessing the weekly group support to address their own weight problems. Thus, a family-based approach to changing shopping and cooking habits, as well as increasing activity levels, would be most appropriate. Some of those parents or guardians who were not already members did join at the same time as their child, whereas a minority of the supporting adults either did not wish to address their own weight or did not (from visual observation) have a weight problem themselves. What was very clear from the qualitative data is that the adult figure often played a very important role in the young person’s weight management journey. For example, an adult having the skills and abilities to facilitate healthier eating for the whole family and having the time to attend weekly was important. The commitment and understanding of such an individual was considered, by all the group facilitators, to be quite pivotal. Those parents or guardians who had particular problems in supporting the young member tended to be those who themselves struggled with their own weight management journey.

A major difficulty is ensuring that parents/guardians only express positive encouragement and facilitate an appropriate home environment that supports weight management. The qualitative evaluation would suggest that a written parental guide to supporting their teenagers’ weight management journey could be helpful, although there is much emphasis on the benefits of a supportive environment within the group sessions.

Only 59% of all health professionals supporting the young member provided any weight management guidance. As a consequence of this evaluative observation, a booklet was produced to both outline the nature of the support that the young person would receive and thus hopefully ensure that the health professional appreciated the evidence based nature of the support. This booklet (Slimming World, 2007) contains a simple form and directional guide for the health professional to sign alongside the age-specific BMI chart. It is also suggested within the booklet that the health professional review the young person’s BMI goal every 3 months to encourage collaboration between the young person, their health professional and the group facilitator. The aim for the partnership proforma is also to minimise any insensitivity in the tone of the recommendation that may have been read by the young person. Regular height measurements are necessary given that this age group potentially experiences natural growth spurts, which in turn influences the BMI measurement, as well as to ensure that any changes in dietary habits do not have a negative effect on linear growth.

Generally, the group facilitators felt either ‘very comfortable’ or ‘comfortable’ about having 11–15 year olds in the group that had previously only been attended by adults. Less than 4% of the facilitators had reservations, which may have simply been a result of the newness of the challenge/concept, and which required slight adaptation to the way the group ran, and particularly the behaviour change techniques employed. Facilitating parents’ support required additional skills, which then may have contributed to the slight uneasiness as to how the young person has fitted into the group because the facilitator was very conscious of the impact that any negativity may have on the young person’s weight loss journey. The responses may be different if the enquiry was repeated now that the facilitators are more familiar with this age group attending the weekly support meetings.

## Limitations

The response rate (of approximately 15%) to the questionnaire was poor and this may add some bias to the data reported. The group facilitators had also not fully appreciated the need for height data to be recorded for this age group and therefore the complete data set for corresponding weight, height and age was reduced.

The 8-month study period ended at the same time as the long summer holiday period and so it is unknown whether those young members returned to group following this period. This may have affected both the attendance and weight change data.

## Conclusions

Childhood obesity is one of the most significant health challenges currently facing the UK and thus requires a number of interventions to be available to support young people to lose weight. The community weight management organisation studied has the capacity (with over 9000 weekly UK weight management groups) to adopt a family-based approach supporting young people aged 11–15 years in managing their weight for as long as they need the support. Approaching 10 000 young people each year have chosen to attend one of these groups and have been successful in moving towards a healthier weight and adopting healthier lifestyles with their families support.

It is important that an evidence-based approach is employed; that on-going evaluation is undertaken to ensure that young people are being empowered to make long-term lifestyle changes; and that there is partnership working with both parents/guardians and health professionals. Subsequent to the present evaluation being undertaken, more quantitative research looking at actual lifestyle changes made by young people has taken place and has recently been reported (Stubbs *et al.*, 2012).

The findings of the present study suggest that 11–15 year olds are quite happy to attend a weight management group primarily attended by adults. The community weight management organisation studied takes a family-based approach and successfully supports young members to manage their weight.

**Conflict of interest, source of funding and authorship**

All of the named authors are employed in some capacity by Slimming World.

All aspects of the data collection were funded by Slimming World.

AA was the lead researcher and CP, JA, JL critically reviewed the manuscript and approved the final version submitted for publication. JS provided statistical advice.
